# Inbreeding depression in *Solanum carolinense *(Solanaceae), a species with a plastic self-incompatibility response

**DOI:** 10.1186/1471-2148-8-10

**Published:** 2008-01-16

**Authors:** Jorge I Mena-Ali, Lidewij H Keser, Andrew G Stephenson

**Affiliations:** 1 Department of Biology, The Pennsylvania State University, University Park, PA 16802, USA; 2 Laboratory of Entomology and Laboratory of Plant Physiology, Wageningen University, Wageningen, The Netherlands; 3 Department of Biology, Amherst College, Amherst, MA 01002, USA

## Abstract

**Background:**

*Solanum carolinense *(horsenettle) is a highly successful weed with a gametophytic self-incompatibility (SI) system. Previous studies reveal that the strength of SI in *S. carolinense *is a plastic trait, associated with particular *S*-alleles. The importance of this variation in self-fertility on the ability of horsenettle to found and establish new populations will depend, to a large extent, on the magnitude of inbreeding depression. We performed a series of greenhouse and field experiments to determine the magnitude of inbreeding depression in *S. carolinense*, whether inbreeding depression varies by family, and whether the estimates of inbreeding depression vary under field and greenhouse conditions. We performed a series of controlled self- and cross-pollinations on 16 genets collected from a large population in Pennsylvania to obtain progeny with different levels of inbreeding. We grew the selfed and outcrossed progeny in the greenhouse and under field conditions and recorded various measures of growth and reproductive output.

**Results:**

In the greenhouse study we found (1) a reduction in flower, fruit and seed production per fruit in inbred (selfed) progeny when compared to outbred (outcrossed) progeny; (2) a reduction in growth of resprouts obtained from rhizome cuttings of selfed progeny; and (3) an increase in the ability to self-fertilize in the selfed progeny. In the field, we found that (1) outcrossed progeny produced more leaves than their selfed siblings; (2) herbivory seems to add little to inbreeding depression; and (3) outcrossed plants grew faster and were able to set more fruits than selfed plants.

**Conclusion:**

*Solanum carolinense *experiences low levels of inbreeding depression under greenhouse conditions and slightly more inbreeding depression under our field conditions. The combined effects of low levels of inbreeding depression and plasticity in the strength of SI suggest that the production of selfed progeny may play an important role in the establishment of new populations of *S. carolinense*.

## Background

In many species of angiosperms, both male and female reproductive structures are found in the same flower. This arrangement is thought to facilitate the deposition and collection of pollen by pollinators in just one visit. However, it also creates the potential for self-fertilization. Self-fertilization is problematic because it increases homozygosity, thereby reducing the contribution of overdominance to fitness and exposing deleterious recessives to selection. As a consequence, selfed progeny tend to suffer from inbreeding depression, i.e. a reduction in fitness of selfed offspring compared to outcrossed offspring [1,2].

Inbreeding depression has been measured for a wide variety of species possessing a range of mating systems [1-8]. These studies have shown that species that typically outcross tend to have greater inbreeding depression than species that typically self [5,7,9-13], that inbreeding depression is usually greater when measured under field conditions than under greenhouse (benign) conditions ([3,12,14-21], but see [22-25]) and that inbreeding depression varies among families within populations (see [9,11,26] for references). Furthermore, several studies have suggested a strong connection among inbreeding, herbivory, and disease [27-33]. In these studies, resistance to herbivores and pathogens was usually found to decrease with inbreeding [30], in most cases in a family-specific manner [27-29,31,33,34]. In fact, it has been proposed that specialist herbivores should perform better on outcrossed plants than on inbred plants [35]. Therefore, field studies regarding the estimation of inbreeding depression should take into account the extent of herbivory as well as the potential family-specific effects of inbreeding on measures of fitness.

Because of its adverse effects on fitness, inbreeding depression has been regarded as a major force in the evolution of plant mating systems [36-38]. Many species of plants have evolved traits that reduce selfing. Such traits include morphological changes in the positioning of the sex structures (herkogamy, enantiostily, [39]), temporal uncoupling of maturation of the male and female parts within flowers (protandry and protogyny [40,41]) and biochemical recognition and rejection of self-pollen (self-incompatibility [42-44]).

Self-incompatibility (SI) is a genetic mechanism, usually controlled by a single highly polymorphic locus called the *S*-locus; each polymorphic variant is referred to as an *S*-allele [45]. SI allows a pistil to recognize and reject self-pollen prior to fertilization, based on biochemical interactions between pollen and pistils [46,47]. In the Solanaceae, SI disrupts the growth of pollen tubes that have an *S*-allele in common with the pistil they pollinate [48,49] thus avoiding fertilization. The disruption of self pollen tube growth is caused by specific ribonucleases (called *S*-RNases) produced by the *S*-alleles in the pistil [50]. These RNases enter the growing pollen tubes, where they degrade messenger and ribosomal RNA of pollen tubes identified as incompatible [51]. This generalized degradation eventually causes tube growth to arrest [48,52] or slow down relative to cross pollen tubes [32].

*Solanum carolinense *L. is a rhizomatous short-lived perennial, native to the eastern United States and Canada. *Solanum carolinense *has a gametophytic SI system (i.e., specific S-RNases are expressed in the pistil and selectively reject self pollen), typical of the Solanaceae [53]. Unlike most self-incompatible plants, however, *S. carolinense *is a weed that inhabits early successional habitats, waste places, crop fields and pastures. It is listed as a noxious weed by the USDA [54] and the Seeds Act and Regulations of Canada [55] and it is classified as an invasive weed in all of the 43 states in which it has been reported. Self-incompatibility is uncommon in weeds and early successional species [56-59] because disturbed habitats require frequent episodes of colonization [hence populations are repeatedly founded by one or a few individuals bearing a limited number of S-alleles, [60,61]), effective population sizes are small (supporting few S-alleles, hence compatible cross pollen may limit fruit and seed production, [62]), and habitats are often short-lived (so there is limited time for the migration of additional S-alleles into populations, [56]).

In previous studies we have investigated this apparent anomaly (i.e., a highly successful weed that is self-incompatible) and we have found that the SI response in *S. carolinense *is a plastic trait – its strength being affected by the age of the flowers [32], and prior fruit production [63] and that there are genetic differences among families in their self-fertility [64,65]. In short, these studies reveal that when outcross pollen is scarce (older flowers remain unpollinated) and/or when few or no outcross fruit are produced on the first 3–5 inflorescences, some plants are capable of setting self seed. We have also recently shown that variability in self-fertility is associated with particular *S*-alleles (i.e., plants carrying certain alleles set significantly more selfed seed than plants not carrying these alleles). The importance of this variation in self-fertility on the ability of horsenettle to found and establish new populations will depend, to a large extent, on the magnitude of inbreeding depression. Inbreeding depression is expected to be high in horsenettle because, as a species with an RNase-mediated GSI response (i.e., active S-RNases are expressed and effectively identify and reject self pollen), selfing would not be that common.

In the study reported here we performed a series of greenhouse and field experiments in order to determine the magnitude of inbreeding depression in *S. carolinense*, to determine whether inbreeding depression varies by family and to determine whether the estimates of inbreeding depression vary under field and greenhouse conditions in horsenettle.

## Results

In this study, we assessed the extent of inbreeding depression in 6 inbred (selfed) and 6 outbred (outcrossed) progeny from 16 genets of *Solanum carolinense *under both greenhouse and field conditions. The percentage of seed germination varied from 65 to 95%. There was no significant effect of Cross (type of pollination [self or cross] that produced the seed) on the percentage of germinated seeds (mean ± SE: Self seed = 81.9 ± 3.9%; Outcrossed seed: 79.7 ± 3.7%; *t *= 0.41; *df *= 15; *p *= 0.68). In the greenhouse study, selfed progeny set significantly fewer seeds following outcross pollinations than outcrossed progeny (Tables 1, 2). Although initial measures of growth did not differ between selfed and outcrossed progeny (e.g., days to flower, Table 1), the selfed progeny showed a decrease in the vigor of plants (resprouts) produced from rhizome cuttings. These rhizome resprouts produced fewer leaves and were smaller when obtained from selfed progeny (Tables 1, 2) indicating perhaps that fewer resources were allocated to vegetative spread via rhizomes in selfed progeny compared to outcrossed progeny. However, selfed plants were better at setting fruits following self pollinations, with a twofold increase in fruit set compared to the selfed fruit produced on outcrossed progeny. The Index of Self Compatibility (*ISC *= *n*_*self*_/*n*_*outcrossed*_, where *n*_*self *_is the count obtained after self-pollinations and *n*_*outcrossed *_is the count obtained after outcross pollinations; see Methods) indicates that the self progeny seem to have become more self-compatible (Table 1, 2). We also found that Genet (the family of the selfed and outcrossed seeds) has a significant (or nearly significant; 0.05 <*p *< 0.10) effect on many of our measures of vegetative vigor and reproductive output (Table 2). In addition, the analyses of variance also revealed significant Genet by Cross (self or outcross pollination) interactions for many of our measures of reproductive output indicating that the families differ in their magnitude of inbreeding depression.

**Table 1:** Mean ± SE and estimates for inbreeding depression (*d*) for fifteen vegetative and reproductive traits in selfed and outcrossed progeny from the greenhouse experiment. The estimates of *δ *were calculated as 1 minus the proportion of mean values from selfed progeny to the mean values from the outcrossed progeny (see Methods).

	Days to Flower		Staminate Flowers		Perfect Flowers		Total Number of Flowers		Outcross fruit per pollination	
**selfed progeny**	38.2	± 0.4	7.8	± 1.2	200.5	± 8.4	280.0	± 13.9	0.96	± 0.04
**outcrossed progeny**	38.5	± 0.4	6.5	± 1.1	206.2	± 9.1	302.5	± 13.9	1.01	± 0.03
**inbreeding depression**, δ	0.01		-0.20		0.03		0.07		0.04	
	**Outcross seed per fruit**		**Outcross seed per pollination**		**Self fruit per pollination**		**Self seed per fruit**		**Self seed per pollination**	

**selfed progeny**	82.6	± 3.2	79.8	± 3.7	0.44	± 0.04	20.2	± 5.1	11.1	± 2.1
**outcrossed progeny**	94.8	± 3.0	93.9	± 3.5	0.24	± 0.04	20.6	± 4.7	6.9	± 2.0
**inbreeding depression**, δ	0.13		0.15		-0.83		0.02		-0.62	
	**ISC(fruit)**		**ISC(seed)**		**Height at transplant**		**Leaf nodes**		**Leaf length**	

**selfed progeny**	0.40	± 0.03	0.22	± 0.02	3.16	± 0.09	6.4	± 0.2	5.13	± 0.12
**outcrossed progeny**	0.27	± 0.03	0.10	± 0.02	3.40	± 0.09	6.6	± 0.2	5.57	± 0.12
**inbreeding depression**, δ	---		---		0.07		0.04		0.08	

**Table 2:** Mixed model analysis of variance for vegetative and reproductive traits from the greenhouse experiment.

Dependent Variable	Effect	df	*F*	*p*
Days to Flower	Genet	15, 15	3.30	0.0135
	Cross	1, 15	0.28	0.6026
	G × C	15, 150	1.45	0.1310
Staminate Flowers	Genet	15, 15	1.89	0.1139
	Cross	1, 15	0.62	0.4435
	G × C	15, 150	2.02	0.0175
Perfect Flowers	Genet	15, 15	3.64	0.0086
	Cross	1, 15	0.21	0.6526
	G × C	15, 150	2.47	0.0029
Total Number of Flowers	Genet	15, 15	3.84	0.0066
	Cross	1, 15	1.30	0.2717
	G × C	15, 150	2.47	0.0029
Outcross fruit per pollination	Genet	15, 15	0.90	0.5794
	Cross	1, 15	0.81	0.3837
	G × C	15, 149	0.93	0.5326
Outcross seed per fruit	Genet	15, 15	1.34	0.2880
	Cross	1, 15	8.01	0.0127
	G × C	15, 148	1.57	0.0898
Outcross seed per pollination	Genet	15, 15	1.17	0.3818
	Cross	1, 15	7.71	0.0141
	G × C	15, 149	0.97	0.4859
Self fruit per pollination	Genet	15, 15	2.46	0.0455
	Cross	1, 15	13.49	0.0023
	G × C	15, 150	1.94	0.0230
Self seed per fruit	Genet	14, 14	2.12	0.0788
	Cross	1, 20	0.00	0.9529
	G × C	14, 60	0.92	0.5400
Self seed per pollination	Genet	15, 15	2.36	0.0533
	Cross	1, 15	2.15	0.1636
	G × C	15, 149	1.50	0.1109
ISC(fruit)	Genet	15, 15	2.67	0.0333
	Cross	1, 15	8.03	0.0126
	G × C	15, 148	2.01	0.0182
ISC(seed)	Genet	15, 15	3.88	0.0063
	Cross	1, 15	12.40	0.0031
	G × C	15, 148	3.14	0.0002
Height at transplant	Genet	15, 15	2.75	0.0296
	Cross	1, 15	4.02	0.0632
	G × C	15, 345	0.66	0.8201
Number of leaf nodes	Genet	15, 15	1.50	0.2207
	Cross	1, 15	1.15	0.2998
	G × C	15, 345	0.92	0.5465
Leaf length	Genet	15, 15	2.33	0.0565
	Cross	1, 15	6.45	0.0227
	G × C	15, 345	1.02	^0.4318^

After completion of the greenhouse study, two rhizome cuttings from each of the self and cross progeny from each of the 16 original genets were transplanted into two field plots. One plot was sprayed weekly with an insecticide. In this sprayed field plot, outcrossed plants had a significantly greater number of leaf nodes at six weeks after transplanting than selfed progeny (Tables 3, 4). There were also slight but not significant differences in herbivory between selfed and outcrossed plants (Table 4). Curiously outcrossed plants had slightly greater herbivory than selfed plants. The primary herbivores that we observed included the eggplant flea beetles (*Epitrix hirtipennis *and *E. fuscula*), the tobacco hornworm (*Manduca sexta*) and both the Colorado and false potato beetle (*Leptinotarsa decemlineata *and *L. juncta*), all of which are specialists on the Solanaceae and include some of the most important pests of cultivated species in this family. In the nonsprayed plots there was severe herbivory resulting in the death of 25 plants (14 cross progeny and 11 self progeny) and 79% of those plants that did survive failed to flower or set fruit (63 cross progeny and 69 self progeny). For those plants that survived, the outcrossed progeny had a greater number of leaf nodes six weeks after transplanting (*F*_1,15 _= 5.10; *p *< 0.05) and a higher ratio of fruits per flower (*F*_1,8 _= 5.34; *p *< 0.05). In contrast to the greenhouse study, we found no significant effects of Genet (i.e., family) or Genet by Cross interactions on vegetative vigor or reproductive output under field conditions.

**Table 3:** Mean ± SE and estimates for inbreeding depression (*d*) for eight vegetative and reproductive traits in selfed and outcrossed progeny in the sprayed treatment under field conditions. The estimates of inbreeding depression *δ *were calculated as 1 minus the proportion of mean values from selfed progeny to the mean values from the outcrossed progeny. Since measures of herbivory are inversely related to fitness (i.e., higher herbivory levels reflect lower fitness), we estimated inbreeding depression for herbivore resistance as 1-[(1-*h*_*s*_)/(1-*h*_*x*_)], where *h*_*s *_is the mean level of herbivory for the selfed progeny and *h*_*x *_is the mean herbivory level measured on the outcrossed progeny (see Methods).

	Leaf Nodes (July)		Leaf Nodes (August)		Staminate Flowers		Perfect Flowers	
**selfed progeny**	8.0	± 0.4	31.6	± 1.6	7.2	± 1.1	15.5	± 2.5
**outcrossed progeny**	8.6	± 0.4	40.4	± 1.5	7.4	± 1.1	20.8	± 2.4
**inbreeding depression**, δ	0.07		0.22		0.03		0.26	

	**Total Number of Flowers**		**Number of Fruits**		**Fruits per Flower**		**Herbivory**	

**selfed progeny**	22.9	± 3.2	14.2	± 2.5	0.86	± 0.05	0.30	± 0.04
**outcrossed progeny**	28.6	± 3.2	17.9	± 2.4	0.80	± 0.04	0.39	± 0.04
**inbreeding depression**, δ	0.20		0.21		-0.08		-0.15	

**Table 4:** Mixed model analysis of variance for several vegetative and reproductive traits of sprayed plants grown in the field

Dependent Variable	Effect	df	*F*	*p*
Leaf Nodes (July)	Genet	15, 15	1.05	0.4653
	Cross	1, 15	1.44	0.2487
	G × C	15, 156	0.48	0.9488
Leaf Nodes (August)	Genet	15, 15	1.37	0.2770
	Cross	1, 15	15.79	0.0012
	G × C	15, 156	1.47	0.1212
Staminate Flowers	Genet	15, 15	1.14	0.4027
	Cross	1, 15	0.02	0.8824
	G × C	15, 156	1.38	0.1634
Perfect Flowers	Genet	15, 15	1.46	0.2363
	Cross	1, 15	2.34	0.1469
	G × C	15, 156	1.19	0.2840
Total Number of Flowers	Genet	15, 15	1.46	0.2380
	Cross	1, 15	1.58	0.2282
	G × C	15, 156	1.11	0.3493
Number of Fruits	Genet	15, 15	1.25	0.3345
	Cross	1, 15	1.18	0.2940
	G × C	15, 156	1.15	0.3136
Fruits per Flower	Genet	15, 15	1.87	0.1181
	Cross	1, 16	1.03	0.3263
	G × C	15, 118	1.18	0.2959
Herbivory	Genet	15, 15	0.83	0.6357
	Cross	1, 15	2.28	0.1516
	G × C	15, 156	0.85	^0.6259^

## Discussion

Although *Solanum carolinense *has an RNase-mediated self-incompatibility system [53,66], our previous studies [32,63] have shown that when cross pollen is scarce (older flowers remain unpollinated and/or few or no outcross fruit are developing on a plant), plants are capable of setting some self seed. Whether this plasticity in self-fertility plays a role in colonization and establishment of new populations of horsenettle depends, to a large extent, on the magnitude of inbreeding depression.

Numerous studies have suggested that inbreeding depression is a potent force in the evolution of plant breeding systems and that inbreeding depression is greater for plants that typically outcross than for plants that typically self due, presumably, to the purging of deleterious recessives upon repeated inbreeding, (see reviews by [2,10,27]). In this study, we found that for many of the individual reproductive output measurements there were no significant effects of inbreeding. However, to determine the total effect of inbreeding depression on reproductive output (*δ*), it is necessary to calculate the multiplicative fitness effects of inbreeding across the various components/measurements of reproductive output [16]. This overall value of *δ *can be estimated as 1 - Π(*R*_*i*_), where *R*_*i *_is the relative performance of the selfed progeny for each trait (see Methods). In the greenhouse experiment, the multiplicative effects of inbreeding on total reproductive output (germination percentage [*R *= 1.03] × the total number of flowers per plant [*R *= 0.93] × the number of outcross fruits per pollination [*R *= 0.96)] × the number of seeds per fruit [*R *= 0.87]) is 0.83, indicating that the average inbred progeny suffers a reduction of 17% in reproductive output. Similarly, the multiplicative effects of inbreeding on reproductive output in the field experiment (sprayed plot), flower number per plant (*R *= 0.80) × fruits per plant (*R *= 0.79) is 0.63, indicating that on average, inbred plants suffer a moderate 37% reduction in reproductive output due to inbreeding depression compared to outbred progeny (at least under our field conditions). In contrast to other predominately outcrossing species, the overall impact of inbreeding on reproductive output in *S. carolinense *is low (greenhouse estimate of *δ *= 0.17) or moderate (field estimate of *δ *= 0.37) [see [2,11] and references therein].

Other studies have also found that inbreeding depression increases under field conditions compared to the greenhouse and more benign field conditions (e.g., plants frequently watered and fertilized, [3,12,14-16,19-21,67]). Because field estimates of inbreeding depression include the combined impacts of inbreeding depression and natural enemies (herbivores and pathogens) on fitness, natural enemies that preferentially attack inbred plants will inflate the estimate of inbreeding depression (because both reduce fitness) while natural enemies that preferentially attack outbred plants will reduce the estimate of inbreeding depression. In this study, we found more (although not significantly more) herbivory on outcrossed progeny than on selfed progeny. It was our intention to have a low herbivory treatment (sprayed weekly) and a moderate herbivory treatment (no spray). However, the no spray treatment experienced uniformly severe levels of herbivory: 25 plants died and 79% of the plants that did live failed to reproduce. This is probably due to the fact that cultivated tomato, potato and eggplant were growing in nearby fields and are grown year after year at our field site (an agricultural experiment station), causing the specialist herbivore community to artificially build up over the years. Consequently, the large numbers of specialist herbivores present in our site resulted in a high herbivory and a severe herbivory treatment. In general, specialist herbivores are thought to prefer feeding on outbred plants over inbred plants, because these herbivores are adapted to locate their hosts based on their volatile compounds produced by the plants and to feed on the secondary chemicals present in outbred plants [35,68]. The opposite would be true for generalist herbivores that prefer feeding on S plants because inbreeding can directly compromise plant defense mechanisms and the general reduction in plant vigor associated with inbreeding may prolong vulnerable stages of development [35,67,69,70]. Even if natural enemies show no preference for either inbred or outbred plants, the impact of severe herbivore pressure (such as that found in our field treatments) on fitness can mask more subtle differences in vigor between self and outcrossed progeny. The heavy herbivory that we observed in our field plots reveals why *S. carolinense *is a particularly problematic weed in and around Solanaceous crop fields – it serves as a reservoir for specialist insects that can potentially re-colonize agricultural fields.

In the greenhouse, we also found significant effects of Genet (i.e., family) on our various measures of vegetative vigor and reproductive output indicating that there is broadsense heritability for traits related to fitness in our population. Perhaps, more importantly for this study, we found significant Genet by Cross (i.e., inbred or outbred progeny) interactions indicating that the amount of inbreeding depression varies by family. These findings suggest that our Genets differed in the number and type of deleterious recessives/overdominant loci that affect reproductive output. Moreover, we found that the selfed progeny set significantly more fruits upon self-pollination than did the outcrossed progeny. Our previous studies have found that all of the plants that we examined produced at least some self seed when self pollinations are made on older flowers and on plants with few or no developing outcrossed fruits [32,63] and that the Genets that produce the most seeds under these conditions possess certain *S*-alleles (i.e., leaky *S*-alleles, [65]). Because the selfed progeny on these plants can inherit the leaky *S*-allele through either the pollen and the ovule, the selfed progeny are more self-fertile (on average) than the outcrossed progeny from the same plant.

In the greenhouse, the vegetative vigor of rhizome sprouts from outcrossed plants was greater than that of selfed plants, indicating that inbreeding depression can potentially affect vegetative spread in this species under field conditions. In horsenettle the above-ground parts die soon after the first frost of the autumn while the below-ground parts overwinter and new shoots emerge early in the spring [55]. These plants send out rhizomes that spread up to 1 m from the original plant and thereby ease in the invasion and spread of newly colonized areas. The reduced vigor of rhizome sprouts from selfed plants may limit their ability to compete with outcrossed plants in established populations. However, the ability to self-fertilize when population sizes are small may be particularly important for this weedy species that lives in ephemeral habitats and experiences frequent episodes of colonization and extinction. Under these conditions, the increased ability of selfed progeny to self-fertilize (compared to the outcrossed progeny) makes them more likely to produce and disperse seed in newly colonized habitats where outcross pollen may limit seed production due to a low diversity of *S*-alleles.

## Conclusion

Genetic variants that promote self-fertilization should increase in frequency, due to the inherent 50% transmission advantage and the ability to produce offspring when cross pollen is scarce, unless these variants are opposed by other evolutionary forces such as inbreeding depression and pollen discounting [1,71,72]. Our findings reveal that the population-wide estimates of inbreed depression in *S. carolinense *are low under greenhouse conditions and moderate under our field conditions. Moreover, we found that inbreeding depression varies significantly across Genets for most of our measures of vegetative vigor and reproductive output and that selfed progeny are more self fertile than outcrossed progeny. Taken together, these results suggest that the genetic variants underlying plasticity in the SI system should increase in frequency (especially when they are found in association with Genets with low levels of inbreeding depression) and that plasticity in the SI system could play a role in the establishment of new populations of this important weed. However, further studies are needed to determine if our findings are robust throughout the extensive geographical range and the variety of habitats occupied by *S. carolinense *in order to determine if horsenettle has a stable mixed mating system (with low self fertility) or whether the mating system has taken the first steps toward more complete self fertility.

## Methods

### Plant material

*Solanum carolinense *L. is a weedy, herbaceous perennial that is found in ephemeral habitats and agricultural fields throughout southeastern Canada and central and eastern United States [73]. Once established it spreads via horizontal rhizomes that can extend over 1 m from the parent stem [74], easing in the invasion and spread of newly colonized areas [55]. The above-ground parts die soon after the first frost of the autumn, marking the end of both the flowering and fruiting season. The below-ground parts overwinter and new shoots emerge early in the spring. Both growth and reproduction are indeterminate. The flowers are approx. 3 cm in diameter, with 5 partially fused white to violet petals; five stamens with short filaments and large, fused yellow anthers (6–9 mm long) that surround the exerted pistil. The flowers are visited by pollen-gathering bees, which must vibrate the flowers to remove pollen from the poricidal anthers [53]. Inflorescences consist of 1–20 flowers that mature acropetally. The fruit is a globose berry, smooth and glabrous, yellow or orange at maturity, 10–20 mm in diameter, and typically contain 60–100 seeds [55]. The majority of the flowers are perfect and functionally hermaphroditic. However, some of the flowers, usually located at the tip of the raceme, exhibit reduced non-functional pistils and are considered functionally staminate [75].

Horsenettle plants were collected from a large population located near State College, Pennsylvania. Rhizome cuttings were taken from 20 plants in the field that were located at least 5 m. apart, in order to decrease the possibility of taking rhizomes from the same genet. These cuttings were brought to the greenhouse, planted in 1-gallon pots, allowed to resprout, grow and flower. After flowering, we cut the stems off and moved the pots to a cold room set at 4°C to vernalize for 6–8 weeks. After the cold treatment, the pots were returned to the greenhouse and allowed to acclimate for a week. We then created ramets from each of the 20 plants (genets) by dividing the rhizome into 5–6 pieces of similar size. Each rhizome cutting was replanted in a 1-gallon pot and allowed to resprout and grow. Four of the ramets were used in the controlled pollination experiment (see below), and the remaining ramets were returned to the coldroom. All of the ramets from two of the original 20 genets failed to resprout and therefore could not be used in this study.

### Controlled pollinations on the parental generation

We divided the four ramets per genet into two groups. We performed only outcrossed pollinations on two ramets and only self-pollinations on the other two ramets. On both self-only ramets and both cross-only ramets per genet, we performed the assigned (i.e., self or outcross) pollinations every 3–4 d (flowers typically last 5–7 d in the greenhouse) on every flower that opened until a total of 40 flowers per ramet were pollinated. The outcrossed pollinations were performed by collecting pollen in a microcentrifuge tube from at least five different genets using a buzz-pollination device (a modified electric toothbrush); vibrating the tube to thoroughly mix the pollen; and then touching the mixture to a stigma. Self-pollinations were made in the same manner except that pollen was collected from 2–3 flowers on the same plants as the flowers to be pollinated. All pollinations were performed in a greenhouse that is approved for growth of transgenic plants – hence the windows are equipped with netting so that bees cannot enter the greenhouse even when the windows are open for ventilation. Moreover, because pollen is released by the anthers only upon vibration (buzzing by bees) it is unlikely that interior greenhouse pests (such as white flies and thrips) could gather and transfer pollen. At maturity (approx. 6 weeks) the fruits were collected and the number of mature seeds produced per fruit was recorded; the seeds were air-dried for 1–2 d and then stored in plastic vials with some desiccant. Two of the 18 genets used in this experiment did not produce enough flowers to complete all pollinations and were therefore excluded from this study. All 16 remaining genets produced at least 20 selfed seeds from the two selfed ramets combined.

### Greenhouse experiments using selfed and outcross progeny

In order to determine the presence and the extent of inbreeding depression in *Solanum carolinense*, we used the progeny obtained from the controlled pollinations. For each of the 16 genets, we sowed 20 outcrossed and 20 selfed seeds in plastic trays in the greenhouse and allowed them to germinate; we recorded the number of days to germination and the total number of seeds that germinated. After the first true pair of leaves was produced, we randomly selected 6 outcrossed and 6 selfed seedlings per genet and planted them in 1-gallon pots. These pots were distributed on greenhouse benches in a randomized block design, with one plant per cross per genet in each block (for a total of six blocks). We recorded the number of days to first flower and the number of perfect and staminate flowers produced by each plant one day per week. Because flowers last 5–7 days in the greenhouse, these counts underestimate total flower production. Consequently, at the termination of flowering, we harvested the inflorescences and counted the number of flower scars. We also performed a series of self pollinations on 5–7 flowers of each plant and allowed these flowers to set fruits. Two to three weeks after the completion of the self pollinations, we performed a series of cross pollinations on 5–7 flowers of each plant. (Note: previous studies [63] had indicated that self pollinations would not set fruit if similar aged fruits from cross pollinations were already developing on the plant although additional cross pollinations would set fruit). Together, the self and cross pollinated flowers represented only a small fraction of the total flowers produced on each plant. Consequently, resources were unlikely to limit fruit and seed production. At maturity, we collected the fruits and counted the number of seeds in each fruit. We calculated the index of self-compatibility (ISC) for (i) the number of fruits per pollination, and (ii) the number of seeds per fruit using the formula *ISC *= *n*_*self*_/*n*_*outcrossed*_, where *n*_*self *_is the count obtained after self-pollinations and *n*_*outcrossed *_is the count obtained after outcross pollinations; an ISC value of 1 indicates complete self-compatibility, whereas an ISC of 0 corresponds to complete self-incompatibility [76]. In summary, we were able to calculate the ISC for both the outbred and the inbred progeny from each of the original 16 genets.

To test for the effects of maternal Genet (16 original plants) and Cross (inbred or outbred progeny) on our measures of growth and reproduction, we performed a mixed model ANOVA (proc MIXED, [77]) with Cross as a fixed effect and block, Genet and the interaction between Genet and Cross as random effects. The measures of growth and reproduction included the days to first flower, the number of perfect, staminate and total flowers, the number of fruits per self and per cross pollination, the number of seeds per fruit, the number of seeds per self and per cross pollination, the ISC values from fruit set and seed set, the number of leaf nodes on rhizome cuttings prior to transplanting into the field (see below), and the height of the rhizome resprouts prior to transplanting into the field (see below). All proportion variables were arcsine (square root) transformed prior to analysis. We calculated estimates of inbreeding depression for all these variables as the mean value obtained from selfed progeny divided by the mean value from the outcrossed progeny. We then estimated overall inbreeding depression (*δ*) under greenhouse conditions as the multiplicative combination of the relative performance of selfed progeny from seed germination, flower production, fruit set and seed production using the formula 1 - Π(*R*_*i*_), where *R*_*i *_= *X*_*self*_/*X*_*outcross*_) is the relative performance of the selfed progeny for each trait, *X*_*self *_is the mean value for each trait in the selfed progeny and *X*_*outcrossed *_is the mean value for each trait in the outcrossed progeny.

### Field experiments

In order to determine if the estimate of inbreeding depression differs under field conditions, we made rhizome cuttings from each of inbred and outbred progeny from each of the 16 maternal plants (Genets) used in the greenhouse experiment. Three equal sized (approximately 7.5 cm long) cuttings per progeny were obtained and sown into 1-liter pots. These cuttings were allowed to resprout and grow in the greenhouse for three weeks. We then recorded the plant height and the number of leaves. We randomly selected two ramets per cross per genet and transplanted them into two 15 m × 30 m experimental plots at The Pennsylvania State University Agricultural Experimental Station at Rock Springs, PA in the summer of 2005. Each plot contained six progeny per Cross per Genet (6 selfed progeny + 6 outcross progeny × 16 genets = 192 plants); these ramets were distributed randomly within the plot. One of the two plots was sprayed weekly with Asana XL (Dupont), a contact insecticide. The other plot was not sprayed. We recorded the number of leaf nodes at one week and six weeks after transplanting, the number of perfect and staminate flowers once per week, and the number of fruits set following natural (open) pollination. Because each flower last for 5–7 days under field conditions the flower counts are an unbiased underestimate of total flower production. It was our intention to determine total flower production by counting flower scars on inflorescences at the termination of flowering as we did in the greenhouse study. However, heavy herbivory on several plants prevented us from doing so. We assessed the level of herbivory by recording the amount of herbivore damage on the 4 youngest leaves on each plant once every two weeks; we measured damage per leaf area using a qualitative scale from 0–5, with 0 being no herbivory and 5 being more than 75% of leaf area removed by herbivores. To determine the occurrence of inbreeding depression under field conditions, we performed a mixed model ANOVA (proc MIXED, [77]) with Cross as a fixed effect and Genet and the interaction between Genet and Cross as random effects. We applied this model to the number of leaf nodes after transplant, the number of perfect and staminate flowers, the total number of flowers, the total number of fruits, the number of fruits per perfect flower and the mean level of herbivory. All proportion data were arcsine(square root) transformed prior to analysis. We calculated estimates of *R*_*i *_for all these variables (excluding herbivory) following the method described in the greenhouse study. In the case of herbivory, we estimated the value of *R *for herbivore resistance as [(1-mean herbivory in the selfed progeny)/(1-mean herbivory in the outcrossed progeny)]; this correction was done because a smaller value of herbivory (and therefore a higher value of herbivore resistance) is considered a higher estimate of fitness. We estimated the overall value of *δ *on reproductive output under field conditions (sprayed plot) as the multiplicative effects of flower and fruit production using the same formula described for the greenhouse study. Because of high mortality and the failure of a large number of plants to reproduce in the unsprayed plot, plants in the sprayed and unsprayed treatments were analyzed separately.

## Authors' contributions

JIM collected and prepared the parental population, participated in the design of the study and in the collection of greenhouse and field data, performed the statistical analysis and drafted the manuscript. LHK performed the greenhouse and field data collection and helped draft the manuscript. AGS conceived of the study, participated in its design and coordination and helped draft the manuscript. All authors have read and approved the final manuscript.
